# The vascular function effects of adding exenatide or meal insulin to basal insulin therapy in early type 2 diabetes

**DOI:** 10.1186/s12933-023-01781-z

**Published:** 2023-03-09

**Authors:** Ravi Retnakaran, Jiajie Pu, Chang Ye, Alexandra Emery, Caroline K. Kramer, Bernard Zinman

**Affiliations:** 1 Leadership Sinai Centre for Diabetes, Mount Sinai Hospital, Toronto, Canada; 2 Division of Endocrinology, University of Toronto, 60 Murray Street, Suite L5-025, Toronto, ON M5T 3L9 Canada; 3 Lunenfeld-Tanenbaum Research Institute, Mount Sinai Hospital, Toronto, Canada

**Keywords:** Endothelial function, Peripheral arterial tonometry, Reactive hyperemia, GLP-1, Endo-PAT

## Abstract

**Objective:**

Basal insulin glargine has a neutral effect on cardiovascular risk in type 2 diabetes (T2DM). In practice, basal insulin is often paired with a glucagon-like peptide-1 receptor agonist (GLP1-RA) or meal insulin; however, the cardiovascular implications of these combinations have not been fully elucidated. In this context, we sought to evaluate the vascular function effects of adding the GLP1-RA exenatide or meal insulin lispro to basal glargine therapy in early T2DM.

**Methods:**

In this 20-week trial, adults with T2DM of < 7-years duration were randomized to 8-weeks treatment with (i) insulin glargine (Glar), (ii) glargine + thrice-daily lispro (Glar/Lispro), or (iii) glargine + twice-daily exenatide (Glar/Exenatide), followed by 12-weeks washout. At baseline, 8-weeks, and washout, fasting endothelial function was assessed with reactive hyperemia index (RHI) measurement by peripheral arterial tonometry.

**Results:**

At baseline, there were no differences in blood pressure (BP), heart rate (HR) or RHI between participants randomized to Glar (n = 24), Glar/Lispro (n = 24), and Glar/Exenatide (n = 25). At 8-weeks, Glar/Exenatide decreased systolic BP (mean − 8.1 mmHg [95%CI − 13.9 to − 2.4], p = 0.008) and diastolic BP (mean − 5.1 mmHg [− 9.0 to − 1.3], p = 0.012) compared to baseline, with no significant changes in HR or RHI. Notably, baseline-adjusted RHI (mean ± SE) did not differ between the groups at 8-weeks (Glar 2.07 ± 0.10; Glar/Lispro 2.00 ± 0.10; Glar/Exenatide 1.81 ± 0.10; p = 0.19), nor did baseline-adjusted BP or HR. There were no differences between the groups in baseline-adjusted RHI, BP or HR after 12-weeks washout.

**Conclusion:**

Adding either exenatide or lispro to basal insulin therapy does not appear to affect fasting endothelial function in early T2DM.

*Trial Registration:* ClinicalTrials.Gov NCT02194595.

## Introduction

The 2008 Food and Drug Administration (FDA) guidance requiring ascertainment of the cardiovascular safety of new glucose-lowering medications has launched an era in which the vascular implications of diabetes therapies are fundamental considerations in the management of type 2 diabetes (T2DM)[1, 2]. In this regard, glargine is unique amongst older (i.e. pre-2008) diabetes therapies in having been the subject of a dedicated cardiovascular outcome trial that confirmed its neutral impact on vascular risk [3]. In current clinical practice, basal insulin is often complemented with either a glucagon-like peptide-1 receptor agonist (GLP1-RA) or meal insulin [4–6]. However, in the absence of assessment with a dedicated cardiovascular outcome trial, the vascular implications of these combinations are less certain and have generally been inferred from post-hoc analyses of previous trials evaluating their components [7, 8].

In the recently-reported PREserVing Beta-cell Function in Type 2 Diabetes with Exenatide And InsuLin (PREVAIL) Trial, we demonstrated that 3 glargine-based regimens (glargine alone, glargine with thrice daily meal insulin lispro, and glargine with twice daily administration of the GLP1-RA exenatide) had similar effects on both pancreatic beta-cell function after 8 weeks of treatment (primary outcome) and rates of diabetes remission after 12-weeks washout [9]. Recognizing the opportunity to also evaluate the vascular impact of these therapies, the design of the trial included serial assessment of endothelial function at baseline, after the 8-week intervention, and after washout. Thus, in this context, we now report the vascular function effects of adding exenatide or lispro to basal insulin therapy in early T2DM.

## Methods

PREVAIL was a 20-week, open-label, parallel-arm trial wherein patients aged 30–80 years with T2DM of ≤ 7 years duration were randomized (1:1:1) to 8-weeks treatment with insulin glargine, glargine + thrice-daily lispro, or glargine + twice-daily exenatide, followed by 12-weeks washout. The study protocol and primary outcome have been described in detail previously [9]. The study was approved by the Mount Sinai Hospital Research Ethics Board and registered at ClinicalTrials.Gov (NCT02194595). All participants provided written informed consent. While the primary and metabolic outcomes have been reported recently [9], the current report presents the pre-specified ancillary outcome of endothelial function and associated vascular measures.

## Intervention

The study intervention has been described in detail [9]. In brief, participants were randomly assigned by computer-generated random allocation sequence to 8-weeks treatment with one of the following regimens:

(I) Glargine, administered at bedtime, with doses titrated to target fasting glucose ≤ 5.3 mmol/l;
(II) Glargine at bedtime with thrice-daily pre-meal lispro, with doses titrated to target fasting glucose ≤ 5.3 mmol/l and 2-h postprandial glucose < 8 mmol/l;
(III) Glargine at bedtime with twice-daily exenatide before breakfast and dinner at doses of 5 ug twice daily for the first 4 weeks, followed by 10ug twice daily for the next 4 weeks (glargine titrated to target fasting glucose ≤ 5.3 mmol/l).

The assigned intervention was stopped at 8-weeks, after which participants entered a 12-week washout during which they were advised to follow healthy lifestyle practices for managing T2DM [9].

### Vascular measures

At each of the study visits at baseline, 4-weeks, 8-weeks and washout, heart rate and blood pressure were measured in a seated position, after 10 min of rest, with an automated blood pressure monitor. Two measurements were performed 10 min apart, with the average recorded. At the study visits at baseline, 8-weeks and washout, endothelial function was assessed by peripheral arterial tonometry (PAT) with the Endo-PAT2000 device (Itamar Medical Inc, Framingham, MA). This assessment was performed after overnight fasting (with glargine held that night). On the morning of the testing, meal insulin and exenatide were not administered because of the intention to assess the primary outcome of the trial (beta-cell function) on oral glucose tolerance test (OGTT) in the absence of these medications [9]. Because it was done prior to the OGTT at the study visits, PAT assessment was performed in the fasting state. For this assessment, PAT finger probes were placed on the distal index fingers of supine participants to enable measurement of baseline pulse amplitude. The measurement was then repeated at 30 s intervals for 4 min following deflation of an occluding blood pressure cuff that was placed on the proximal forearm at 60 mm Hg above systolic blood pressure for 5 min. Pulse amplitude data was recorded electronically and analyzed by the Endo-PAT digital signal processing algorithm, which calculated the Reactive Hyperemia Index (RHI) from the ratio of the average post-deflation pulse amplitude to the baseline pulse amplitude, divided by the corresponding ratio in the contralateral control finger (i.e. this hand did not face an occluding blood pressure cuff). Lower RHI has been shown to predict atherosclerotic coronary artery disease [10, 11].

The COVID-19 pandemic prevented completion of the Endo-PAT portion of the protocol in the full study population of 102 participants, first by preventing some in-person visits and later because of unavailability of the PAT finger probes. Accordingly, Endo-PAT assessment was completed in 73 participants, yielding the study population for the current analysis.

### Statistical analyses

Statistical analyses were conducted with R 4.2.1 and on an intention-to-treat basis. Two-tailed *P* values < 0.05 were considered statistically significant. Continuous variables were tested for normality of distribution, and natural log transformation of skewed variables were conducted where necessary. Characteristics of the study arms at baseline were compared by Analysis of Variance (normally distributed variables) or Kruskal–Wallis test (skewed variables), or either Chi-Square test or Fischer exact test (categorical variables) (Table 1). Paired *t-*tests were conducted to assess changes in vascular measures from baseline to 8-weeks within each treatment arm (Table 2). The longitudinal changes in vascular measures from baseline to 8-weeks were compared between the 3 treatment groups by generalized estimating equation (GEE) model, wherein the treatment effect and time effect were examined. Vascular outcomes after intervention and after 3-month washout were compared between the groups by Analysis of Covariance (ANCOVA) with adjustment for their baseline measurements (Table 3).

**Table 1:** Baseline characteristics of the 3 treatment groups: (i) glargine, (ii) glargine + lispro, and (iii) glargine + exenatide

	Glargine	Glargine	Glargine	
		+ lispro	+ exenatide	
	(n = 24)	(n = 24)	(n = 25)	p
Age (years)	56.4 (9.5)	60.6 (7.1)	55.5 (8.9)	0.09
Sex (% male)	14 (58)	10 (42)	17 (68)	0.17
Ethnicity				0.40
White n(%)	15 (62.5)	16 (66.7)	18 (72)	
South Asian n(%)	2 (8.3)	1 (4.2)	4 (16)	
Other n(%)	7 (29.2)	7 (24.1)	3 (12)	
Duration of diabetes (years)	3.5 (2.1)	4.1 (2.0)	3.4 (2.0)	0.43
DM medications before study				0.91
Lifestyle only n(%)	3 (12.5)	2 (8.3)	5 (20)	
Metformin n(%)	17 (70.8)	16 (66.7)	15 (60)	
DPP-4 inhibitor n(%)	1 (4.2)	0 (0)	0 (0)	
SGLT-2 inhibitor n(%)	0 (0)	0 (0)	1 (4)	
Sulfonylurea n(%)	0 (0)	1 (4.2)	0 (0)	
Metformin + DPP-4 inhibitor n(%)	3 (12.5)	4 (16.7)	3 (12)	
Metformin + SGLT-2 inhibitor n(%)	0 (0)	1 (4.2)	1 (4)	
Metformin + Sulfonylurea n(%)	0 (0)	0 (0)	0 (0)	
Vascular complications				
Retinopathy n(%)	0 (0)	0 (0)	0 (0)	*
Albuminuria n(%)	1 (4)	1 (4)	1 (4)	1.00
Neuropathy n(%)	1 (4)	2 (8)	2 (8)	1.00
Cardiovascular disease n(%)	1 (4)	2 (8)	1 (4)	0.84
Vascular Risk Factors				
Hypertension n(%)	10 (42)	19 (79)	12 (48)	0.02
Hypercholesterolemia n(%)	12 (52)	14 (58)	16 (64)	0.69
Current smoking n(%)	2 (14)	3 (18)	3 (13)	1.00
Cardioprotective medications				
ACE inhibitor/ARB n(%)	9 (38)	16 (67)	12 (48)	0.12
Statin n(%)	14 (58)	15 (62)	17 (68)	0.78
Aspirin n(%)	4 (17)	7 (29)	3 (12)	0.32
Body mass index (kg/m2)	32.2 (7.0)	31.2 (5.7)	30.9 (5.8)	0.75
Waist circumference (cm)	108 (15.6)	104 (13.7)	105 (12.5)	0.59
Baseline A1c (%)	6.6 (0.7)	6.3 (0.7)	6.6 (0.7)	0.34
Vascular measures				
Systolic blood pressure (mmHg)	133 (18.7)	135 (15.2)	137 (15.5)	0.69
Diastolic blood pressure (mmHg)	82 (9.7)	81 (9.0)	84 (10.7)	0.56
Heart rate (beats per min)	71 (13.0)	72 (11.8)	69 (9.5)	0.62
RHI	1.88 (0.41)	1.83 (0.46)	1.83 (0.32)	0.86

Continuous variables are presented as mean followed by standard deviation in parentheses (if normal distribution) or median followed by interquartile range (if skewed distribution). Categorical variables are presented as proportions

**Table 2:** Changes in vascular measures from baseline to 8-weeks in response to the interventions

Vascular measures	Glargine			Glargine + Lispro			Glargine + Exenatide		
	mean	95%CI	P	mean	95%CI	P	mean	95%CI	P
Systolic BP (mmHg)	− 3.7	(− 10.2 to 2.7)	0.24	− 2.3	(− 9.0 to 4.5)	0.50	− 8.1	(− 13.9 to − 2.4)	0.008
Diastolic BP (mmHg)	− 3.7	(− 7.9 to − 0.5)	0.08	− 0.5	(− 3.9 to 2.8)	0.74	− 5.1	(− 9.0 to − 1.3)	0.012
Heart rate (bpm)	0.0	(− 3.8 to 3.9)	0.99	0.2	(− 4.5 to 4.9)	0.92	0.7	(− 2.3 to 3.8)	0.62
RHI	0.2	(− 0.04 to 0.44)	0.10	0.17	(− 0.14 to 0.47)	0.27	− 0.03	(− 0.14 to 0.08)	0.58

P-values reflect comparison between measurement at 8-weeks and measurement at baseline

**Table 3:** Baseline-adjusted vascular outcomes after intervention and after 3-month washout

	Glargine	Glargine	Glargine	p
		+ Lispro	+ Exenatide	
Vascular outcomes after 8-week intervention				
Baseline-adjusted systolic BP at 8-weeks (mmHg)	130 (2.7)	133 (2.7)	128 (2.6)	0.38
Baseline-adjusted diastolic BP at 8-weeks (mmHg)	79 (1.7)	81 (1.7)	78 (1.7)	0.34
Baseline-adjusted heart rate at 8-weeks (bpm)	71 (1.6)	72 (1.7)	71 (1.6)	0.91
Baseline-adjusted RHI at 8-weeks	2.07 (0.10)	2.00 (0.10)	1.81 (0.10)	0.19
Vascular outcomes after 3-month washout				
Baseline-adjusted systolic BP at 20-weeks (mmHg)	133 (2.1)	132 (2.0)	128 (1.9)	0.25
Baseline-adjusted diastolic BP at 20-weeks (mmHg)	83 (1.6)	81 (1.5)	79 (1.5)	0.28
Baseline-adjusted heart rate at 20-weeks (bpm)	70 (1.6)	72 (1.6)	69 (1.5)	0.53
Baseline-adjusted RHI at 20-weeks	1.74 (0.10)	1.87 (0.09)	1.96 (0.09)	0.25

Data are presented as adjusted mean (SE)

## Results

Table 1 shows the baseline characteristics of the study participants randomized to (i) insulin glargine alone (Glar; n = 24), (ii) glargine + pre-prandial lispro (Glar/Lispro; n = 24), and (iii) glargine + twice-daily exenatide (Glar/Exenatide, n = 25), respectively. There were no significant differences between the groups in clinical, metabolic or vascular measures, apart from a higher prevalence of hypertension in the Glar/Lispro group (p = 0.02). Of note, there were no differences between the groups in blood pressure (BP), heart rate and RHI (Table 1).

**Fig. 1:**
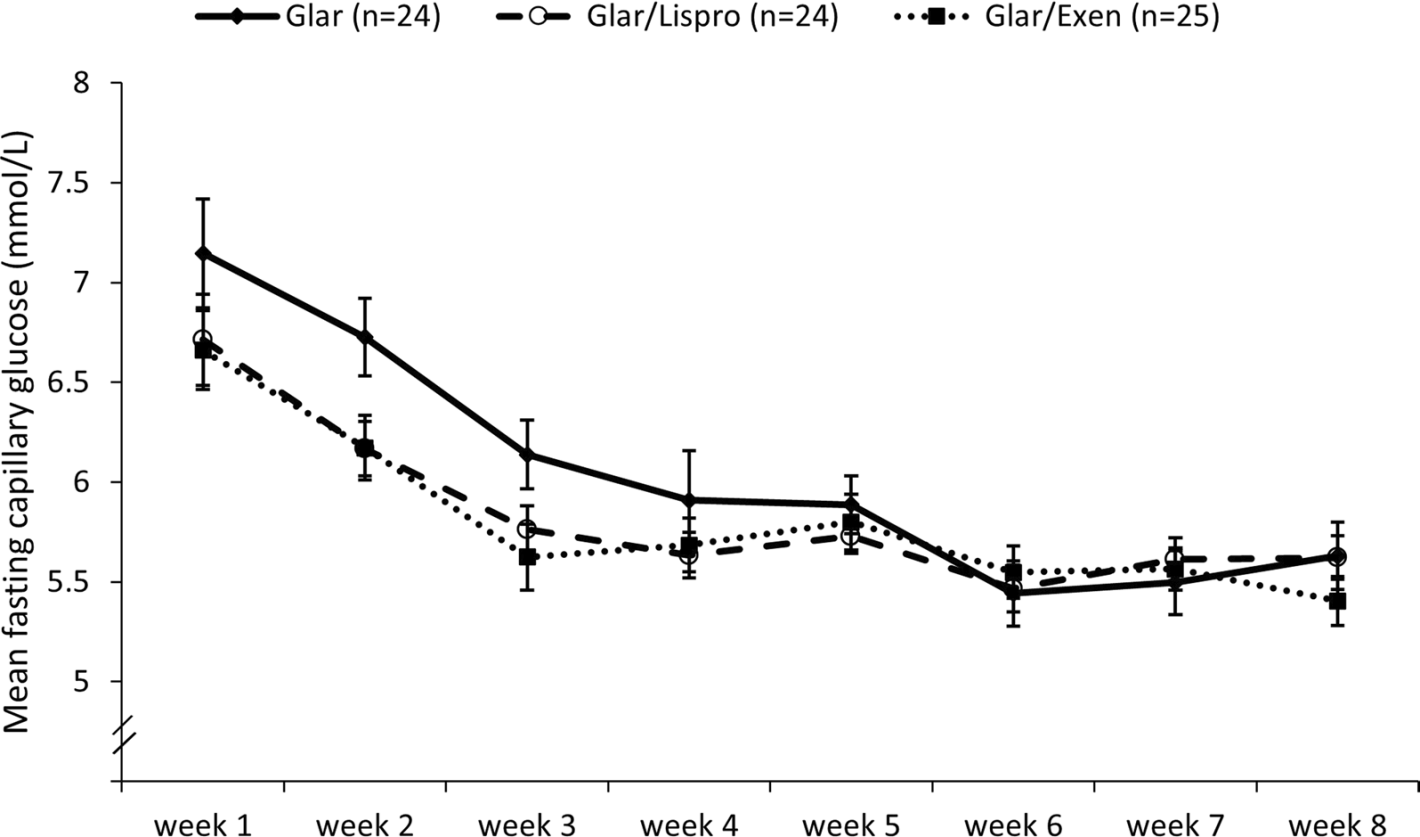
Mean fasting capillary glucose on self-monitoring during each week of therapy in the 3 treatment arms (data are presented as mean with standard error)

**Fig. 2:**
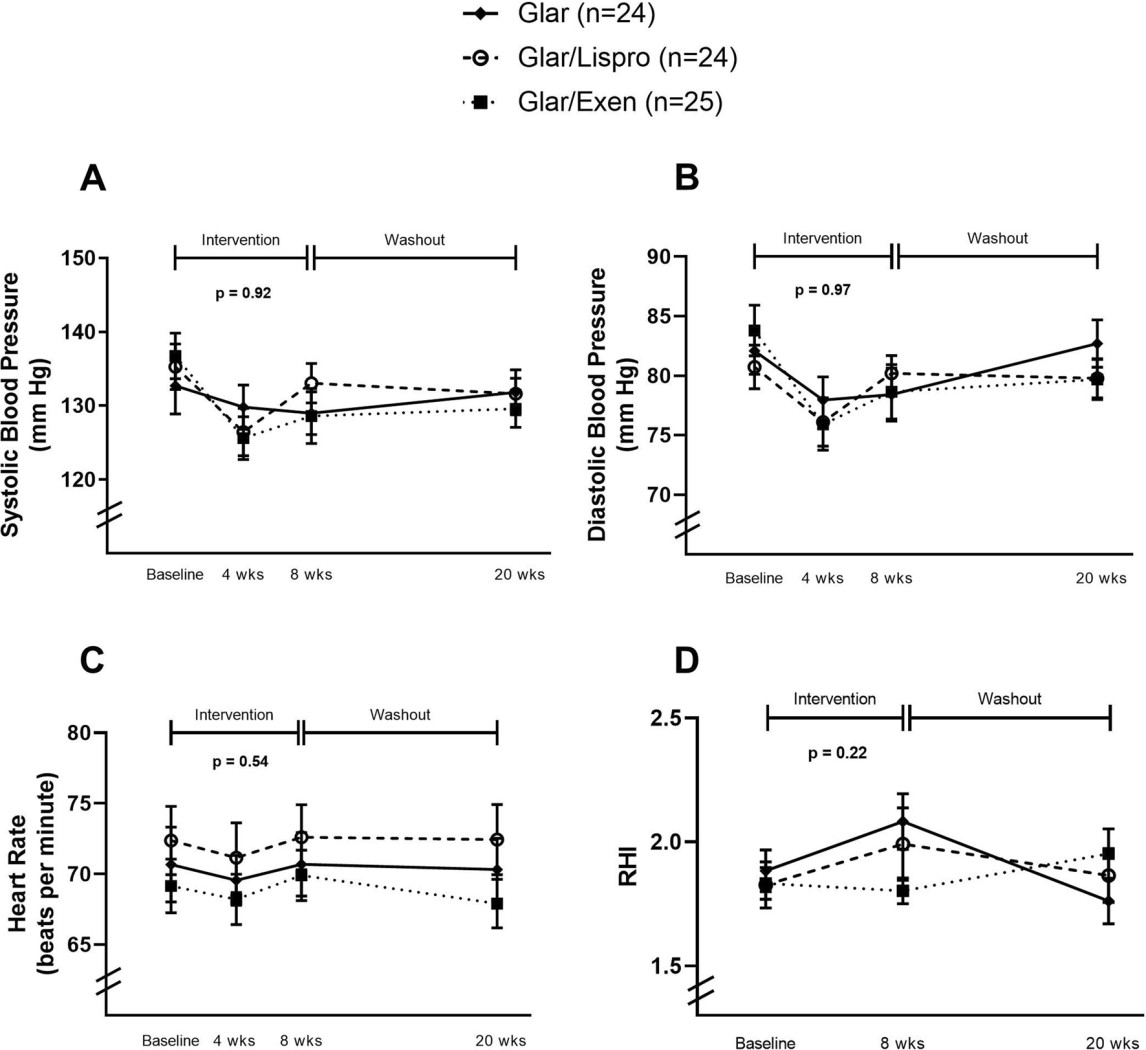
Vascular measures in each of the treatment arms during the trial: **(Panel A)** systolic blood pressure; **(Panel B)** diastolic blood pressure; **(Panel C)** heart rate; and **(Panel D)** reactive hyperemia index (RHI)

Figure 1 shows the similar improvement in mean fasting capillary glucose in the 3 groups across the 8 weeks of intervention. As in the overall trial [9], baseline-adjusted A1c at 8-weeks was lowest in Glar/Exenatide followed by Glar/Lispro and Glar (mean 5.79% vs 5.85% vs 6.16%; p = 0.0002), as was baseline-adjusted BMI (mean 30.9 vs 31.6 vs 32.1 kg/m^2^; p < 0.0001). After 8-weeks of therapy, Glar/Exenatide decreased systolic BP (mean − 8.1 mmHg [95%CI − 13.9 to − 2.4], p = 0.008) and diastolic BP (mean -5.1 mmHg [-9.0,-1.3], p = 0.012), while there no significant BP changes in the Glar and Gar/Lispro groups (Table 2). There were no significant changes in heart rate and RHI between baseline and 8-weeks in any of the 3 groups (Table 2). Importantly, as shown in Fig. 2, there were no differences in (A) systolic BP, (B) diastolic BP, (C) heart rate or (D) RHI between the 3 groups at either 4-weeks or 8-weeks, nor were there differences between the groups in the changes over time in these measures during the 8-week intervention (systolic BP: p = 0.92; diastolic BP: p = 0.97; heart rate: p = 0.54; RHI: p = 0.22). Indeed, at 8-weeks, the pre-specified ancillary outcome of baseline-adjusted RHI (mean ± SE) did not differ between the groups (Glar 2.07 ± 0.10; Glar/Lispro 2.00 ± 0.10; Glar/Exenatide 1.81 ± 0.10; p = 0.19), nor did baseline-adjusted BP or heart rate (Table 3). Similarly, at the washout visit at 20-weeks, the baseline-adjusted vascular measures also did not differ between the groups (Table 3).

## Discussion

In this study, 8-weeks of glargine/exenatide combination therapy decreased BP in patients with T2DM of modest duration, without inducing significant changes in heart rate or RHI. Notably, there were no differences in baseline-adjusted RHI between glargine alone, glargine + lispro and glargine + exenatide after the 8-week intervention or after subsequent 12-weeks washout. These data thus suggest that adding either exenatide or lispro to basal insulin therapy does not affect endothelial function in early T2DM.

For determining the cardiovascular risk implications of a glucose-lowering regimen, the optimal evaluation would be provided by a dedicated cardiovascular outcome trial. Though such trials have shown neutral effects of both glargine [3] and exenatide once weekly [12] on cardiovascular risk, combination therapy has not been prospectively assessed in this way. The knowledge gap arising from the absence of such a trial is underscored by work from Ceriello et al. demonstrating that the simultaneous infusion of insulin and GLP-1 acutely improves endothelial function in patients with T2DM more than either therapy on its own [13]. These data thus raise the question of how chronic basal insulin/GLP1-RA combination therapy may impact vascular function.

To date, there has been limited investigation of this question. In a recent study of 34 glargine-treated patients with T2DM with mean A1c ~ 8.0% at baseline, the addition of either once daily lixisenatide or insulin glulisine (administered only before breakfast) for 8-weeks was found to yield similar effects on systemic hemodynamic measures in the fasted state (including BP, HR, stroke volume, cardiac index, systemic vascular resistance index, and normalized augmentation index) [14]. The current study builds upon that report, but with salient design differences in (i) evaluating more than twice as many participants (n = 73) with well-controlled T2DM, (ii) providing prandial insulin at all 3 meals in the lispro group, and (iii) including a third arm with glargine alone. With this design, we show that adding either twice-daily exenatide or thrice-daily lispro to glargine therapy for 8-weeks did not affect RHI, BP or HR.

Our data suggest that, in the setting of basal insulin therapy, the postprandial metabolic coverage provided by exenatide and lispro does not improve endothelial function. However, this interpretation should be coupled with certain caveats. First, with well-controlled T2DM of modest duration and relatively high rates of adoption of cardioprotective medications (renin-angiotensin system blockers, statins), it is possible that this study population may not have had sufficiently advanced vascular disease for demonstration of a therapeutic effect on endothelial function or had insufficient dysfunction for such a demonstration at this sample size. Indeed, at baseline, the mean RHI values in each group (ranging from 1.83 to 1.88) exceeded the threshold that typically reflects abnormal function (< 1.67). Second, since RHI was assessed in only 73 of the 102 participants, it is possible that there was insufficient statistical power to detect differences in this measure. Third, the absence of differences in RHI under fasting conditions (as measured herein) may not exclude differences in endothelial function in the postprandial state. Of note, previous studies have shown that a single dose of exenatide can improve RHI when the latter is measured after a meal challenge [15, 16]. Also, while lower RHI has been shown to predict atherosclerotic coronary artery disease [10, 11], it remains to be established whether improvement in RHI in response to intervention reduces future risk of cardiovascular events.

Another consideration is that, in current practice, long-acting GLP1-RAs have surpassed short-acting formulations in popularity (both with and without basal insulin). Interestingly, a recent study in 112 individuals with T2DM showed that the long-acting GLP1-RA dulaglutide (which has been previously shown to reduce the risk of major adverse cardiovascular events in T2DM [17]) improved RHI after 9-months of treatment but that this effect was not apparent at 3-months [18]. Accordingly, in the current study, the absence of an impact on RHI after 8-weeks may not rule out the possibility of beneficial effects on vascular function emerging with longer duration of therapy.

In conclusion, 8-weeks of glargine/exenatide combination therapy decreased BP, without significant changes in heart rate or RHI. Importantly, RHI at fasting did not differ between glargine alone, glargine + lispro, and glargine + exenatide either after 8-weeks of treatment or after subsequent washout. These data thus suggest that the prandial metabolic coverage provided by adding either exenatide or lispro to basal insulin therapy does not appear to affect fasting endothelial function in early T2DM.

## Data Availability

De-identified data can be made available under restricted access from the corresponding author, for academic purposes, subject to a material transfer agreement and approval of the Mount Sinai Hospital Research Ethics Board.

## Abbreviations

BP: Blood pressure
GLP1-RA: Glucagon-like peptide-1 receptor agonist
HR: Heart rate
PAT: Peripheral arterial tonometry
RHI: Reactive hyperemia index
